# Elicitation of Stress-Induced Phenolic Metabolites for Antimicrobial Applications against Foodborne Human Bacterial Pathogens

**DOI:** 10.3390/antibiotics10020109

**Published:** 2021-01-23

**Authors:** Ashish Christopher, Dipayan Sarkar, Kalidas Shetty

**Affiliations:** Department of Plant Sciences, North Dakota State University, Fargo, ND 58108, USA; ashish.christopher@ndsu.edu (A.C.); dipayan.sarkar@ndsu.edu (D.S.)

**Keywords:** antimicrobials, elicitation, foodborne pathogens, plant phenolics, pentose phosphate pathway

## Abstract

Foodborne bacterial pathogens in consumed foods are major food safety concerns worldwide, leading to serious illness and even death. An exciting strategy is to use novel phenolic compounds against bacterial pathogens based on recruiting the inducible metabolic responses of plant endogenous protective defense against biotic and abiotic stresses. Such stress-inducible phenolic metabolites have high potential to reduce bacterial contamination, and particularly improve safety of plant foods. The stimulation of plant protective response by inducing biosynthesis of stress-inducible phenolics with antimicrobial properties is among the safe and effective strategies that can be targeted for plant food safety and human gut health benefits. Metabolically driven elicitation with physical, chemical, and microbial elicitors has shown significant improvement in the biosynthesis of phenolic metabolites with antimicrobial properties in food and medicinal plants. Using the above rationale, this review focuses on current advances and relevance of metabolically driven elicitation strategies to enhance antimicrobial phenolics in plant food models for bacterial-linked food safety applications. Additionally, the specific objective of this review is to explore the potential role of redox-linked pentose phosphate pathway (PPP) regulation for enhancing biosynthesis of stress-inducible antibacterial phenolics in elicited plants, which are relevant for wider food safety and human health benefits.

## 1. Introduction/Background: Food Safety and Public Health Challenges Related to Bacterial Foodborne Pathogens

Food safety is an essential need coupled to food security and must be addressed for effective solutions to address nutritionally linked global food insecurity challenges. Much of the nutritionally and human health relevant fresh plant foods such as fruits, vegetables, and whole grains are highly susceptible to microbial contamination during pre-harvest cultivation as well as post-harvest transportation, storage, and processing stages [1]. Different factors at pre-harvest production stages such as agronomic practices, environmental conditions, load of biotic and nutritional stressors in plant rhizosphere and phyllosphere, and potential exposure to other abiotic stresses can contribute to the microbial contamination of food plants [1]. Therefore, improvement in food safety of nutritionally relevant plant foods is extremely important to address both infectious diseases related to foodborne pathogens as well as to develop healthy plant-based diets to counter nutritional insecurity related chronic public health challenges. The most common bacterial foodborne pathogens responsible for wider foodborne illness outbreaks in plant foods with previous origins in animal foods are *Escherichia coli*, *Salmonella* spp., *Campylobacter* spp., and *Listeria* spp. [2] and will be the focus of this review. The bacterial-mediated spoilage and deterioration due to contamination of fresh and processed plant foods not only lead to serious foodborne illnesses but also cause significant post-harvest losses of nutritionally relevant plant foods, which concurrently affects nutritional insecurity challenges worldwide. Therefore, it is essential to explore novel and effective strategies that can help counter these bacterial pathogens for improving the safety of grains, vegetables, and fruits that are at risk of contamination at any stage during the farm-to-fork chain of food production, post-harvest storage, and distribution. Furthermore, improving food safety of nutritionally relevant and phenolic bioactive-enriched fresh foods such as fruits, vegetables, whole grains, medicinal, and culinary herbs is also relevant to address rapid emergence of diet-linked non-communicable chronic diseases (NCDs) such as type 2 diabetes, obesity, and cardiovascular diseases [3,4]. 

Overall, the increasing occurrence and cases of foodborne diseases worldwide can be attributed to environmental degradation, increasing human population growth, urbanization, the expansion in international food trade, and rapid industrialization of food production and processing. Beyond its impact on human health, foodborne diseases also adversely affect the economic growth of countries, due to loss of income during illness, the recall of contaminated food products, the damage to the credibility of food manufacturers, and the financial losses endured by countries because of the rejection of food imports or exports based on suspected contamination of foods [5]. To address such serious and ever-increasing food safety challenges, specifically to reduce the incidence in outbreak of foodborne illnesses, several scientific innovations and policy measures such as improved rapid detection tools, safety certification, novel product formulation, better packaging, improved preservation, regulation, compliance, and regular monitoring were advanced in recent decades. However, even with such advancements, 1 in 10 people worldwide fall ill after eating contaminated foods, and 420,000 people die from such illness every year [6]. According to the Centers for Disease Control and Prevention (CDC, Atlanta, GA, USA), 1 in 6 Americans fall sick, while 128,000 are hospitalized and 3000 die every year due to foodborne illness in the United States [7]. The economic burden of foodborne illness in the United States is around 15.6 billion US dollars each year [8]. Furthermore, with globalization of trade and vast transportation of agricultural produce across borders and within countries, the food supply chain becomes more complex and therefore possesses greater risk of serious disease outbreak from contaminated foods.

Among major foodborne diseases, the most frequent causes of foodborne illness were due to diarrheal disease infections from norovirus and the bacterial pathogen *Campylobacter* spp. that accounted for 120 million and 96 million annual cases respectively in the year 2010 [9,10]. In the United States, scientists and public health professionals were able to estimate annual number of cases of foodborne illnesses, hospitalizations, and deaths from the years 1998 to 2008, which were attributed to 17 food commodities under three major groups such as aquatic animals (fish, crustaceans, and mollusks), land animals (dairy, eggs, beef, game, pork, and poultry), and plants (grains/beans, oils/sugars, fruits/nuts, fungi, leafy vegetables, roots, sprouts, and vines) [11]. The estimates obtained in this study showed a total of 9.6 million annual illnesses, of which around 51% (~4.9 million), 42% (~4.0 million), and 6% (~600,000) were due to consumption of contaminated plant, land animal, and aquatic animal commodities, respectively. Among the 17 food commodities, leafy vegetables were found to have the highest number of cases associated with foodborne illnesses (22%) followed by dairy (14%), fruits/nuts (12%), and poultry (10%) [11]. Additionally, there have been cases of foodborne illnesses due to the consumption of contaminated sprouts of alfalfa, clover, and beans, which were responsible for 53 outbreaks, 1876 illnesses, and 209 hospitalizations between the years 1998 and 2016 [10].

Some of the microbial contamination risk factors of the above plant foods that exist in the farm-to-fork food chain include irrigation water, manure or compost, livestock or wild animal fecal contamination, field and worker sanitation, and cross-contamination during post-harvest preservation, storage, transportation, and distribution [12,13]. The use of contaminated manure/compost, soil, and irrigation water (surface or ground water) can in turn contaminate the seeds, roots, and surfaces of fresh produce such as lettuce, spinach, basil, berries, onions, melons, tomatoes, and sprouts [14]. Some of the common bacterial pathogens associated with foodborne illnesses due to consumption of contaminated fresh produce include *Aeromonas*, *Bacillus*, *Clostridium*, *E. coli* O157:H7, *Listeria*, *Shigella*, *Vibrio*, and *Campylobacter* [13]. Several intrinsic and extrinsic factors such as motility of the pathogen, water activity/humidity, biofilm formation, plant tissue damage, leaching of nutrients, and interactions with other microorganisms within the plant phyllosphere and rhizosphere can determine the susceptibility of fresh plant produce to bacterial contamination [15]. Good agricultural practices (GAP) and good handling practices (GHP) can help minimize the microbial food safety hazards of fresh and highly perishable fruits and vegetables [16]. The World Health Organization (WHO) recommends five key practices to follow in order to reduce bacterial contamination of fresh fruits and vegetables during planting, growing, harvesting, and storing, and these key recommendations include practicing good personal hygiene, protecting fields from animal fecal contamination, using treated fecal waste (manure), evaluating and managing risks from irrigation water, and keeping harvest and storage equipment clean and dry [17]. However, cases of foodborne illnesses linked to bacterial contamination of plant-based foods continue to persist despite the implementation of recommended guidelines and safe agricultural practices that are aimed at reducing this risk. Furthermore, gut-associated pathogens such as *Salmonella* spp., *Enterococcus* spp., *E. coli*, *C. jejuni,* and *Clostridium difficile* have the potential to emerge as superbugs with enhanced morbidity and mortality, as well as with increasing resistance to a broad range of antibiotic therapies [18]. These current challenges warrant a need to look at novel strategies that can help reduce the burden or risk of bacterial contamination of plant-based and nutritionally relevant foods with known human gut-associated pathogenic bacteria, as well as to address the increasing resistance of foodborne bacterial pathogens against common antibiotic drugs. 

In this context, innovations in new antimicrobial strategies must provide multilayered barriers against bacterial contaminations of foods, especially plant-based fresh foods. Stimulation of the host endogenous protective defense response related to the biosynthesis of plant secondary metabolites with antimicrobial function is a novel approach that can provide multi-layered protections to plant-based foods against bacterial contamination. In general, plant secondary metabolites or phytochemicals are a broad range of bioactive compounds that help the plant to adapt to different types of abiotic and biotic stress, and these stress-inducible compounds also display a broad spectrum of growth-inhibitory activity against several bacterial, viral, and fungal pathogens [19]. Therefore, metabolic regulation involving biosynthesis of stress-inducible phenolic antimicrobials can be recruited as a safe and effective strategy to improve the safety of plant-based and nutritionally relevant fresh foods and to reduce the risk of contamination with foodborne bacterial pathogens. Among different strategies, metabolically driven elicitation using physical, chemical, and microbial elicitors can be targeted to stimulate the biosynthetic pathways that are involved in the production of stress-inducible phenolic metabolites with antimicrobial activity, thereby concurrently improving the biosafety of fresh plant-based foods. Based on this rationale, the primary objective of this review is to explore recent developments in the potential benefits of different elicitation strategies to improve biosynthesis of antimicrobial phenolics in nutritionally relevant plant foods. Additionally, the role of such metabolically driven elicitation strategies to stimulate the redox-linked pentose phosphate pathway (PPP) for enhancing the biosynthesis of food safety and human health relevant phenolic metabolites in plant-based foods is also discussed. 

## 2. Phenolic Metabolites of Plants

Under exposure to biotic and abiotic stresses, plants produce a diverse group of secondary compounds with antimicrobial potential. These secondary metabolites can be broadly classified into three main groups, which are terpenoids, alkaloids, and polyphenols, with diverse biological functions relevant for human health and food safety benefits [20]. Several secondary metabolites from plants and plant-based foods such as flavonoids, alkaloids, terpenes, and phenolic acids have shown significant antimicrobial properties. The most common plant-derived compounds with significant antimicrobial properties are mostly phenolic metabolites, which have been traditionally used in food preservation and therapeutic applications since ancient times [21]. The biosynthesis of these phenolic antimicrobial compounds is part of the plant’s natural inducible adaptive responses against external stresses, specifically to counterattack pathogens and insects and to minimize damages induced by abiotic stress factors [19,22]. 

In general, plant phenolics are broadly divided into simple phenolics that include phenolic acids (hydroxycinnamic acids and hydroxybenzoic acids) and coumarins, and polyphenols that include flavonoids (flavones, flavonols, isoflavonols, flavanones, flavanols, anthocyanins, and chalcones) and non-flavonoids (tannins, lignans, and stilbenes) [23]. These stress-inducible phenolic metabolites have diverse biological functions that are involved in plant–insect, plant–microorganism and plant–plant interactions and provide protection to the plant against biotic and abiotic stresses [24]. 

Previously published studies reported that thymoquinone, rutin, epicatechin, myricetin, ellagic acid, and benzoic acids are most effective phenolic compounds against several foodborne bacterial pathogens such as *E. coli*, *Salmonella*, *C. jejuni*, *Listeria monocytogenes,* and *Vibrio parahaemolyticus* [25,26]. In addition to antimicrobial activity, plant phenolic compounds also have other human health relevant pharmacological properties such as antioxidant, anti-hyperglycemic, and anti-hypertensive functions based on their active role in human metabolism as well as their indirect functions through gut microbe modulation, which potentially help to prevent and manage NCDs such as type 2 diabetes or hypertension [3,4,22,27,28]. These pharmacologically relevant secondary metabolites can also be used as valuable medicines, cosmetics, dyes, insecticides, flavoring agents, and fragrances, and as functional ingredients to modulate the organoleptic and nutritional properties of food [3,20,24]. The pharmacological properties of these plant secondary metabolites, specifically phenolics, lies in their ability to interact with molecular or cellular targets that include enzymes, hormone receptors, cell membrane transport proteins, and other protein or DNA targets. Additionally, plant secondary metabolites can alter gene expression and protein biosynthesis, among other vital cellular activities, and can exhibit antimicrobial functions through signal transduction processes or modulation of cellular targets [29]. 

## 3. Role and Mechanism of Phenolic Metabolites as Antimicrobials

The biosynthesis of stress-inducible and antimicrobial phenolics and other secondary metabolites is part of a plant’s natural adaptive response to counterattack pathogens and insects as well as to mitigate damages induced by constantly varying environmental conditions and related abiotic stress factors [19,22]. Exposure to several abiotic and biotic stresses can upregulate the expression of genes that code for key enzymes involved in the phenolics and anthocyanin biosynthetic pathway such as phenylalanine ammonia-lyase (PAL), chalcone synthase (CHS), chalcone isomerase, and flavonoid-O-glucosyltransferase [30,31]. Furthermore, exposure to abiotic or biotic stress results in the hardening or priming of plants helping them to quickly adapt to future stress events. Several mechanisms by which plants are able to do this is through the accumulation of signaling proteins and transcription factors and/or through epigenetic changes that alter gene expression through DNA modification without changes to the genetic code and which result from exposure to the stress [32]. Protective phenolic metabolites that help plants to adapt to different abiotic and biotic stresses as well as their antimicrobial efficacy and potency depend upon the structure–function mechanism of these compounds and the extraction method used to obtain these compounds from food or medicinal plants [33,34]. 

The antimicrobial activity of stress-inducible phenolics is due to their ability to function as inhibitors in microbial biosynthetic pathways such as DNA, protein and cell wall synthesis, as chelating agents that reduce the availability of micronutrients for bacterial growth, and as hydrophobic molecules that permeabilize or destabilize the bacterial cell membrane, all of which can help inhibit the growth of pathogenic Gram-negative and Gram-positive bacteria [19,33,35,36,37,38]. Several phenolics were found to permeabilize the cell membrane of lactic acid bacteria *Oenococcus oeni* and *Lactobacillus hilgardii,* and the hydroxycinnamic acids (*p-*coumaric, caffeic, and ferulic acid) gave a higher ion leakage and proton influx in the damaged bacterial cells when compared to the hydroxybenzoic acids (*p-*hydroxibenzoic, protocatechuic, gallic, vanillic, and syringic acids) [39]. 

Among diverse phenolic compounds, higher antimicrobial activity was found with phenolic acids, flavonoids, tannins, stilbenoids, quinones, and coumarins [33,36]. These phenolics such as flavonoids (flavan-3-ols and flavanols), tannins (condensed or hydrolysable), and non-flavonoid compounds (phenolic acids and lignans) have potential antibacterial, antiviral, and antifungal activity, while non-flavonoid compounds generally have a weaker antimicrobial activity than flavonoids. More specifically, these stress-inducible phenolics display antibacterial activity against a number of human bacterial pathogens such as *Vibrio cholerae*, *Streptococcus mutans*, *C. jejuni*, *E. coli*, *Bacillus cereus*, *Helicobacter pylori*, *Staphylococcus aureus*, *Salmonella*, *Clostridium perfringens*, *L. monocytogenes*, *Pseudomonas aeruginosa,* and *Mycobacterium tuberculosis* [19,33,40,41]. Extracts of a variety of medicinal plants, herbs, spices, fruits, and plant-based foods have been analyzed for their antimicrobial activity against human bacterial pathogens (Table 1) [42,43,44,45,46,47,48,49,50,51,52,53,54]. Likewise, various pure phenolic compounds have also been analyzed for their antimicrobial activity against human bacterial pathogens (Table 2) [35,42,55,56,57,58]. A previous study analyzed 29 Finnish plants for their antimicrobial activity, and it was found that the extracts of meadowsweet (*Filipendula ulmaria* (L.) Maxim.), willow herb (*Epilobium angustifolium* L.), cloudberry (*Rubus chamaemorus* L.), and raspberry (*Rubus idaeus* L.) were most effective against human bacterial pathogens [42]. The same study reported that the pure phenolic compounds such as flavone, quercetin, and naringenin showed mild to strong antimicrobial activity against *S. aureus*, *Staphylococcus epidermis*, *B. subtilis*, *Micrococcus luteus*, *E. coli,* and *P. aeruginosa* [42]. The flavonoid quercetin was shown to bind to the 24 kDa fragment of the gyrase B subunit of the bacterial enzyme DNA gyrase, which is responsible for the negative supercoiling of DNA during DNA replication or transcription [59]. The binding of quercetin was found to inhibit the ATPase activity of gyrase B subunit, thereby inhibiting the supercoiling activity of DNA gyrase, which in turn affects DNA or RNA synthesis resulting in bacterial cell death [59]. In another study, catechins present in green tea extracts were found to inhibit the activity of DNA gyrase by binding to the 24 kDa fragment of gyrase B, thereby inhibiting its ATPase activity [60]. 

The same study showed epigallocatechin gallate (EGCG) to have the highest DNA gyrase inhibitory activity followed by epicatechin gallate (ECG) and epigallocatechin (EGC), while epicatechin (EC) had little or no gyrase inhibitory activity [60].

Similarly, gallic acid, caffeic acid, and ferulic acid dissolved in dimethyl sulfoxide to a concentration of 1000 µg/mL was found to show inhibitory activity against *E. coli*, *P. aeruginosa*, *L. monocytogenes,* and *S. aureus,* respectively, while chlorogenic acid showed inhibitory activity only against *E. coli* and *P. aeruginosa* [55]. The phenolic phytochemicals protocatechuic acid, gallic acid, quercetin, and myricetin showed inhibitory activity against *P. aeruginosa* strains, and the antimicrobial activity was attributed to the inhibition of dihydrofolate reductase (DHFR), an important bacterial enzyme involved in the biosynthesis of folic acid, as molecular docking studies have shown these phenolic phytochemicals to be capable of binding to the active sites of DHFR [35,37]. Furthermore, a combination of these phytochemicals with synthetic folic acid inhibitors sulfamethoxazole and trimethoprim resulted in synergistic and additive modes of interaction, respectively [37].

Previous studies have also found that thymoquinone, rutin, epicatechin, and myricetin function as effective antimicrobial phenolics against *E. coli*, while epicatechin, thymoquinone, rutin, and myricetin were more effective against *Salmonella*. Similarly, inhibitory activity of coumarin against *Salmonella* was also reported [61]. Another study found antibacterial activity of benzaldehydes and benzoic acids against *C. jejuni*, *E. coli*, *L. monocytogenes,* and *Salmonella enterica* [25]. Epigallocatechin-gallate (EGCg), the major catechin found in green tea, showed inhibitory activity against tetracycline-resistant *S. aureus* at a minimal inhibitory concentration (MIC) of 100 µg/mL, and a combined treatment of EGCG at a concentration of 50 µg/mL along with tetracycline gave a 128-fold lower MIC against tetracycline-resistant *S. aureus* [58]. In another study, EGCG showed a MIC of 5 µg/mL against *Enterococcus faecalis,* and the antimicrobial activity was attributed to the production of hydroxyl radicals [62]. The same study also showed EGCG to be able to eradicate seven-day-old *E. faecalis* biofilms as well reduce the expression of virulence genes that code for the virulence factors gelatinase, collagen-binding antigen, cytolysin, and serine protease [62]. 

However, the antimicrobial activity of plant extracts varies widely between different plant parts due to the variations in distribution and concentration of antimicrobial phenolic metabolites in different plant tissues. Different parts (stem, leaf, root, and whole plant) of the tomato cultivar Pitenza showed a MIC ranging from 3.1 to 25 mg/mL against the pathogens *Salmonella* Typhimurium, *E. coli* O157:H7, *S. aureus*, and *Listeria ivanovii* [43]. Similar to the antimicrobial function against foodborne bacterial pathogens, phenolics have also shown antibacterial activity against other pathogenic human gut bacteria such as the ulcer-causing bacterial pathogen *H. pylori*. The phenolic-enriched fraction of cranberry juice was found to suppress the growth of *H. pylori* in a dose-dependent manner at 0, 0.1, 0.33, 1, and 3.3 mg/mL concentrations of the extracts, while the juices of phenolic-rich fruits (cranberry, blueberry, and red grape) at a 2% concentration showed growth-inhibitory activity against *H. pylori* when compared to juices of other fruits that have a relatively lower phenolic concentration (white grape, orange, apple, and pineapple) [44]. In another study, gallic acid and catechin were found to inhibit the growth of two *H. pylori* strains in a dose-dependent manner, with gallic acid showing a stronger inhibitory activity than catechin, and a partial additive growth inhibitory effect was observed when gallic and catechin were used in combination [56]. 

Like the function of individual phenolic acids and their combinations, extracts of food and medicinal plants that are rich in diverse phenolic compounds have also shown high antimicrobial activity in in vitro studies. In a previous study, ethanolic extracts of elite phenolic phytochemical producing clonal lines of oregano and the two main phenolic constituents found in oregano (thymol and carvacrol) were analyzed individually for their antimicrobial activity against foodborne pathogen *L. monocytogenes* [47]. The results of this study revealed that oregano extracts significantly inhibited the growth of *L. monocytogenes* at a concentration of 1200 ppm (comparable to 27.8 µg phenolics/mL), while thymol and carvacrol at a concentration of 150–200 ppm were more effective [47]. The antimicrobial activities of oregano and cranberry extracts against *L. monocytogenes* and *V. parahaemolyticus* were analyzed in seafood and meat systems, and it was reported that oregano and cranberry extract combinations at ratios of 3:1 and 1:1 had optimum antimicrobial activity against *L. monocytogenes* and *V. parahaemolyticus,* respectively, and the efficacy of the antimicrobial activity was improved by the addition of lactic acid [45,46]. Similarly, extracts of five culinary and medicinal herbs from the *Lamiaceae* family (sage, thyme, lemon balm, peppermint, and oregano) were analyzed for their phenolic-linked antimicrobial activity against Gram-negative (*Campylobacter coli*, *E. coli,* and *Salmonella infantis*) and Gram-positive (*L. monocytogenes*, *B. cereus,* and *S. aureus*) bacteria [48]. Among these *Lamiaceae* family herbs, sage was also found to have the highest phenolic content and nonflavonoid/flavonoid ratio, as well as the lowest MIC (mg/mL), against these bacterial pathogens [48]. 

In another study, extracts of sage harvested throughout the year showed antimicrobial activity against both Gram-negative (*E.coli*, *S. infantis*) and Gram-positive (*B. cereus*, *S. aureus*) bacteria; however, the samples collected in summer were most effective against Gram-negative bacteria when compared to the sample harvested in autumn-winter period, which indicated the impact of seasonal variations on the content and function of stress-inducible phenolic antimicrobials of culinary and medicinal herbs [49]. In both of these studies, the Gram-positive bacterial pathogens were more susceptible to the antimicrobial activity of the sage extracts, as evident by the overall lower MIC, when compared to the Gram-negative bacteria, which had an overall higher MIC [48,49]. The reason for the difference in susceptibility of Gram-positive and Gram-negative bacteria to the antimicrobial activity of plant phenolics could be due to morphological differences in their cell wall structure. The cell wall of Gram-negative bacteria is made up of a peptidoglycan layer, which is enclosed by an outer membrane made up of lipopolysaccharides, proteins, and phospholipids, while the cell wall of Gram-positive bacteria contains only a thick peptidoglycan layer. The presence of the outer membrane in the cell wall of Gram-negative bacteria can potentially reduce the interaction of plant phenolic compounds with the bacterial plasma membrane and even slow down the uptake of phenolic compounds into the bacterial cell [38,48,52,63,64]. Extracts of several medicinal plants belonging to the families of *Fabaceae* (e.g., *Tamarindus indica*) [50], *Rubiaceae* (e.g., *Morinda citifolia*) [51], *Compositae* (e.g., *Aspilia mossambicensis*) [52], and *Lamiaceae* (e.g., *Ocimum gratissimum*) [53] have also shown antimicrobial activity against a broad range of bacterial pathogens that include *E. coli*, *Salmonella typhi*, *S. aureus*, *Shigella dysenteriae,* and *Proteus* spp., and the antimicrobial activity can be attributed to the alkaloid, flavonoid, tannin, terpene, quinone, and resin content found in the leaves, stems, and roots of these plants [63]. 

Apart from medicinal plants, extracts of common food plants such as five Chinese purple corn hybrids (*Zea Mays* L.) showed growth-inhibitory activity against *Salmonella enteritidis* and *S. aureus* at a concentration of 25 mg/mL, and the growth-inhibitory activity was attributed to high anthocyanin content including cyanidin derivatives that were detected in the corn hybrids [54]. As mentioned earlier, the antimicrobial activity of stress-inducible phenolic metabolites is due to their ability to function as inhibitors in microbial biosynthetic pathways that include DNA, protein and cell wall synthesis, as chelating agents that reduce the availability of micronutrients for bacterial growth, and as hydrophobic molecules that permeabilize or destabilize the bacterial cell membrane, all of which can help to inhibit the growth of pathogenic Gram-negative and Gram-positive bacteria [19,33,35,36,37,38]. Furthermore, plant phenolics can act in synergy with antibiotics in improving the efficacy of treatment against multidrug-resistant (MDR) bacteria, as these compounds act as antibiotic potentiators (e.g., inhibitors of bacterial efflux pumps and destabilization of bacterial cell membrane) and as virulence attenuators (e.g., disruption of bacterial quorum sensing and modulation of host immune system) [64,65]. Therefore, targeting antimicrobial phenolic-enriched plant extracts alone or complementarily with antibiotic drug therapy is a safe and effective strategy to improve the overall safety against foodborne bacterial pathogens and to address several challenges arising from increasing antibiotic resistance of different bacterial pathogens against common antibiotics globally. In this context, understanding and recruiting metabolically driven strategies to stimulate the biosynthesis of stress-inducible phenolics with antimicrobial potential in food and medicinal plants for their food safety benefits has significant merit.

## 4. Biosynthesis of Stress-Inducible Phenolics in Plant Systems

The biosynthesis of plant secondary metabolites, such as stress-inducible phenolic metabolites, occurs via the phenylpropanoid pathway and depends on the production of metabolites that are formed during primary metabolic activities such as the catabolic glycolytic pathway and the anabolic pentose phosphate and shikimic acid pathways [4,20,66,67]. Metabolites produced in the glycolytic and pentose phosphate pathway (PPP) such as phosphoenolpyruvate and erythrose 4-phosphate are utilized in the shikimate pathway for the production of the aromatic acids tryptophan, tyrosine, and phenylalanine, which are ultimately utilized in the synthesis of a wide variety of plant secondary metabolites such as alkaloids, indole glucosinolates, flavonoids, hydroxycinnamic acids, lignin, and lignans [67]. The phenylpropanoid pathway represents the shift from primary to secondary metabolism and is an indispensable pathway for plants due to its role in the production of monolignols that are utilized in lignin biosynthesis, critical for plant structural support, vascular integrity, and resistance to pathogen attack [4,66,67,68]. The first step in the phenylpropanoid pathway is the deamination of phenylalanine to *trans*-cinnamic acid, which is catalyzed by the enzyme phenylalanine ammonia-lyase (PAL), and the isoforms of this enzyme are encoded by a family of genes found in plants designated as *PAL 1–PAL 4* [67]. Then, *trans*-cinnamic acid is converted to *p*-coumarate via ring modification by the enzyme cinnamic acid 4-hydroxylase, and *p*-coumarate is converted to *p*- coumaroyl CoA via sidechain modification by the enzyme 4-coumarate: CoA ligase, and the metabolite *p*- coumaroyl CoA, which is then used in the enzyme-mediated pathways for biosynthesis of flavonoids, phenolic acids, and lignins [67]. Overall, the redox-linked pentose phosphate pathway (PPP) plays a critical metabolic role in the biosynthesis of secondary metabolites as upregulation of this pathway can stimulate the production of protective phenolic compounds with potential antimicrobial function [4,69]. 

## 5. Role of Pentose Phosphate Pathway (PPP) Regulation in Plants for Biosynthesis of Stress-Inducible Phenolic Metabolites

**Figure 1:**
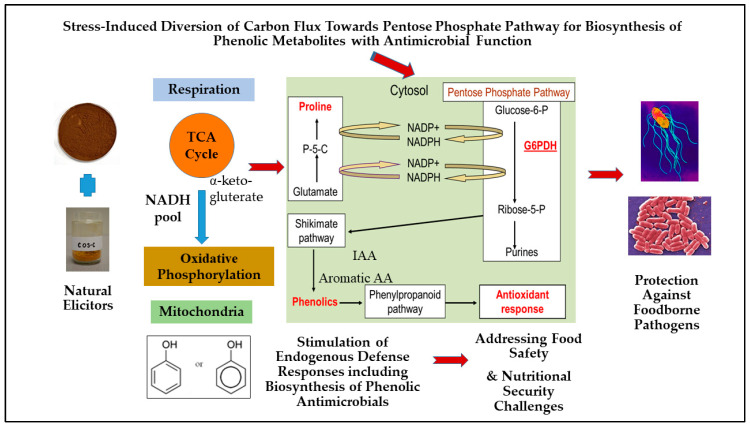
Proposed model of proline-associated pentose phosphate pathway (PAPPP) regulation for biosynthesis of stress-inducible phenolic metabolites in food plants with antimicrobial function. TCA: tricarboxylic acid cycle; NADH: nicotinamide adenine dinucleotide hydrogen; P-5-C: pyrroline-5-carboxylate; IAA: indole acetic acid; AA: amino acids; NADP+: nicotinamide adenine dinucleotide phosphate; NADPH: nicotinamide adenine dinucleotide phosphate hydrogen; Glucose-6-P: glucose 6 phosphate; Ribose-5-P: ribose-5-phosphate.

In the plant system, the pentose phosphate pathway (PPP) generates sugar phosphates, such as erythrose-4-phosphate, which are utilized in the anabolic shikimate and phenylpropanoid pathways to produce phenolic metabolites with diverse protective functions [22]. The initial rate-limiting steps of PPP also generate reducing compounds such as dihydro-nicotinamide adenine dinucleotide phosphate (NADPH_2_), which is critical for diverse anabolic pathways as well as in the antioxidant response pathways [69]. A proposed redox-linked metabolic response model was developed linking proline biosynthesis to the PPP pathway regulation in plants, especially for its relevance in stimulation of biosynthesis of secondary metabolites such as phenolics under abiotic and biotic stress conditions [22,69] (Figure 1). In general, the higher accumulation of proline in plants occurs due to exposure to abiotic stresses, such as low water potential (drought, salinity, or freezing), and subsequent metabolism of proline via its synthesis (cytosol) or catabolism (mitochondria) can play an active role in other redox reactions and energy transfers in plant cells [69,70,71]. Proline synthesis in the cytoplasm or chloroplast involves the conversion of glutamate to pyrroline-5-carboxylate (P5C) and the conversion of P5C to proline, both of which are enzyme-mediated and accompanied by the conversion of NADPH_2_ to NADP^+^ (nicotinamide adenine dinucleotide phosphate) [69,71]. In the proline-associated pentose phosphate pathway (PAPPP) model, the synthesis of proline leads to an increase in the ratio of NADP^+^/NADPH_2,_ which in turn drives the activity of the NADP^+^-dependent enzymes glucose-6-phosphate dehydrogenase (G6PDH) and 6-phosphogluconate dehydrogenase that are involved in the rate-limiting steps of PPP [69,71]. Therefore, upregulation of the proline-linked PPP can enhance the production of erythrose-4-phosphate and help recycle NADP^+^ to NADPH_2,_ which are both utilized in the anabolic pathways for the production of plant secondary metabolites, while NADPH_2_ can also be utilized as a cofactor in the biosynthesis of antioxidants and antioxidant enzymes that include glutathione, superoxide dismutase, catalase, and guaiacol peroxidase [3,67,69]. Furthermore, proline can serve as a reducing equivalent instead of NADH for the synthesis of energy molecules (ATP-adenosine triphosphate) during oxidative phosphorylation in the mitochondria [3,67,69]. This proposed PAPPP model involving biosynthesis of stress-inducible and protective phenolic metabolites is critical for plants to maintain essential metabolic functions and structural integrity under abiotic and biotic stresses [4]. In this context, several biologically based strategies have been advanced to improve biosynthesis of stress-inducible phenolics through modulation of redox-linked PAPPP regulation in food and medicinal plants [22,72].

Elicitation strategies using naturally derived elicitors and other bioprocessed compounds with antimicrobial potential provides an innovative metabolic strategy to stimulate the biosynthesis of stress-inducible phenolic metabolites including antimicrobials through up-regulation of redox-linked PAPPP in food and medicinal plant systems (Figure 1). Several natural and safe compounds have already shown empirical evidence of eliciting phenolic biosynthesis in vitro, primarily such as proline, proline precursors, and proline analogs, fish protein hydrolysate, and soluble chitosan oligosaccharide (COS) [72,73,74,75]. These bioprocessed elicitor compounds either directly act as an antioxidant or stimulate host endogenous protective defense responses by mimicking biotic or abiotic stress induction. Therefore, these elicitors potentially trigger endogenous protective defense responses in plants by up-regulating defense-related anabolic pathways such as protective PAPPP. Previously, seed elicitation using soluble chitosan oligosaccharides (COS) and marine protein hydrolysates were found to improve the phenolic-linked antioxidant and anti-hyperglycemic activities in dark germinated barley and black bean sprouts, and this improvement was attributed to the stimulation of the redox-linked PAPPP and subsequent enhancement in the biosynthesis of the phenolics and associated antioxidant enzyme activity [76,77,78]. The promising results from these previous studies suggested that metabolically relevant elicitation is a safe and effective strategy to enhance biosynthesis of secondary metabolites including stress-inducible phenolics in plant models through stimulation of protective PAPPP, which can be rationally recruited for food safety and relevant applications that benefit human health (Figure 1). 

## 6. Elicitation Strategies to Enhance Stress-Inducible Phenolic Metabolites for Antimicrobial Applications

There is a growing body of published scientific literature on pre- and post-harvest stress-modulating strategies to improve the biosynthesis of phenolic metabolites for diverse food and health benefits, such as targeting the improvement of nutritional, post-harvest preservation, and shelf-life qualities of plant-based foods [4]. However, there is limited research on understanding the role of these strategies to improve inducible phenolics with antimicrobial potential in plant-based foods to address food safety issues arising from the contamination of foods with bacterial pathogens such as *Salmonella*, *Campylobacter,* and *E. coli*. Among different strategies, metabolically driven elicitation is a novel approach to stimulate biosynthesis of inducible and protective phenolics in food and medicinal plants for improving abiotic and biotic stress resilience, as well as to improve nutritional and post-harvest preservation qualities of plant-based foods. Elicitation strategies include the use of elicitors from biological origin (e.g., lipopolysaccharides, proteins, and oregano extracts), chemical origin (e.g., inorganic salts, acetic acid, and silicon), phytohormonal elicitors (e.g., salicylic acid, jasmonic acid, and ethylene.), and physical elicitors (e.g., high pressure, UV radiation, temperature, and wounding) [79,80,81,82], all of which can potentially help to stimulate plant defense responses leading to enhanced biosynthesis of secondary metabolites such as stress-inducible phenolics with antimicrobial potential [47,83,84,85,86,87,88,89,90,91,92,93,94,95] (Table 3). Elicitors can induce a host defense response by binding to specific receptors on plant cells that leads to the activation of signal transduction processes, which occurs in sequential steps that include recognition of elicitor by plant cell receptors; phosphorylation or dephosphorylation of plasma membrane and cytosolic proteins; increase in cytosolic Ca^2+^ content, plasma membrane depolarization, K^+^/Cl^−^ efflux and H^+^ influx, and cytoplasmic acidification; activation of mitogen-activated protein kinase (MAPK); activation of NADPH oxidase and production of reactive oxygen species (ROS); early defense gene expression; phytohormone production (ethylene and jasmonate); late defense gene expression; and accumulation of plant secondary metabolites such as phenolics [96,97]. 

**Figure 2:**
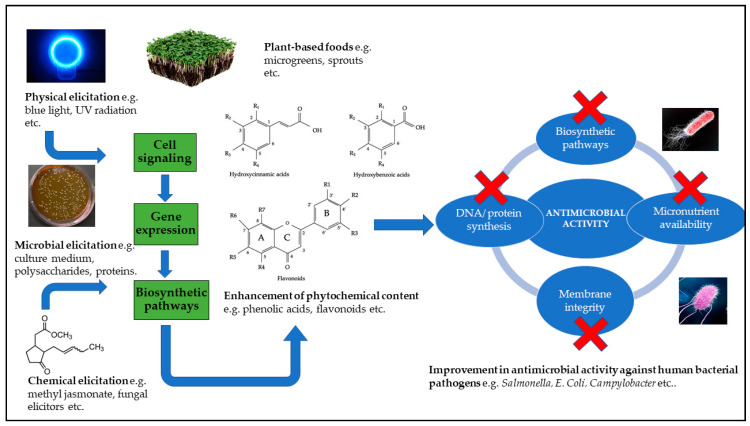
Elicitation strategies to improve phenolic metabolites and associated antimicrobial activity against human bacterial pathogens in plant models.

As mentioned earlier, several studies have shown that elicitation strategies such as exposure to light at different wavelengths and the application of chemical elicitors or phytohormones are capable of inducing a defense response that involves the upregulation of gene expression and the activation and/or enhancement of biosynthetic pathways necessary for the production of phenolic metabolites that have pharmacological properties including potential antimicrobial activity against human gut related bacterial pathogens [79,80,81,82] (Figure 2). Therefore, such a metabolically driven elicitation strategy can be targeted to enhance protective and inducible phenolics for dual functional benefits such as improving human health relevant nutritional qualities and food safety relevant antimicrobial properties in fresh and processed plant foods. 

### 6.1. Physical Elicitation Strategies

Accumulation of phenolic secondary metabolites in plants can occur due to exposure of the plant to different abiotic stress conditions that include temperature stress (heat or cold), water stress (drought or flooding), salinity, radiation (light, UV or ionization radiation), chemical stress (heavy metals, pesticides, or mineral salts), and mechanical stress (wind pressure, wounding, or soil movement) [98]. In previous studies, physical stress elicitors have shown to enhance the phenolic phytochemical content and their bioactivity in several microgreens [99,100,101]. In a study, green and red basil microgreens exposed to different ratios of blue and red light showed a higher fresh biomass and improved chlorophyll synthesis on exposure to a predominant blue light, while a predominant red and blue light improved the phenolic synthesis and antioxidant activity for the green and red basil microgreens, respectively [99]. The same study showed that a predominant blue light gave a 15- and 4-fold increase in rosmarinic and caffeic acid contents, respectively, when compared to white light used as the control [99]. In another study, a combined treatment of blue light (400 nm) with caffeic acid (5 mM) gave a 4 and 1 log (CFU/mL) reduction in the growth of *E. coli* O157:H7 and *Listeria innocua,* respectively, along with an increase in uptake of caffeic acid into the bacterial cells due to cell membrane damage [83]. The same study showed that the leaves of baby spinach inoculated with *E. coli* O157:H7 on the leaf surface had lower growth (log CFU/mL) of *E. coli* O157:H7 when the leaves were sprayed with 5 mM caffeic acid, and the addition of blue light did not significantly enhance the antimicrobial activity of the treatment [83]. Callus obtained from hypocotyl explants of *Brassica nigra* L. cultured under light or dark in vitro conditions were analyzed for their total phenolic content, antioxidant activity, and antimicrobial activity against *E. coli*, *P. aeruginosa*, *K. pneumonia,* and *S. aureus,* and it was observed that the calli grown under light conditions had significantly higher total phenolic content and antioxidant activity (*p* < 0.05), as well as better antimicrobial activity against the bacterial pathogens, when compared to calli grown under dark conditions including the hypocotyl explants that were used as control [84].

Immature radish microgreens treated with hydrogen-rich water (HRW) were exposed to either white light (control), blue light, UV-A radiation, or darkness in order to study the effect of HRW on anthocyanin production and antioxidant activity under different lighting conditions [101]. This study found that blue light with HRW and UV-A with HRW gave 1.12 and 1.17 times higher total phenolics, when compared to treatment with either blue light or UV-A [101]. Additionally, blue light with HRW gave a 1.50- and 1.35-fold higher content of the anthocyanins, cyanidin, and petunidin-3,5-diglucoside, respectively, when compared to the treatment with only blue light, while UV-A with HRW treatment gave a 1.09- and 1.27-fold higher content of cyanidin-3-rutinoside-5-glucoside and cyanidin-3-glucoside, respectively, when compared to treatment with only UV-A [101]. In this study with radish greens, short wavelength light (blue light or UV-A) in combination with HRW enhanced the enzyme activity and upregulated the gene expression levels of the key enzymes involved in anthocyanin biosynthesis such as phenylalanine ammonia-lyase (PAL), chalcone synthase (CHS), chalcone isomerase, and flavonoid-O-glucosyltransferase, and it also improved the antioxidant activity in terms of OH. and O_2_.- radical scavenging activity [101]. 

In general, anthocyanins such cyanidin-3-glucoside, cyanidin-3-rutinoside, and petunidin-3-glucoside found in berries (e.g., cranberry, elderberry) and fruits (e.g., pomegranate, strawberry) have shown growth-inhibitory activity against Gram-positive (*L. monocytogenes*, *S. aureus*, *B. subtilis*, *E. faecalis*, and *C. perfringens*) and Gram-negative *(E.coli*, *P. aeruginosa*, *C. jejuni*, *H. pylori*, *S. enterica* sv. Typhimurium, *S. enterica* sv. Infantis, and *Corynebacterium diphtheriae*) bacterial pathogens [102]. Additionally, many bacterial pathogens are highly sensitive to blue light (400–470 nm) due to the accumulation of naturally occurring photosensitizers such as porphyrins and flavins [103]. Therefore, non-white light elicitor treatment alone or in combination can not only directly inhibit the growth of some bacterial pathogens attached to the surface of the fresh produce, but it can also potentially upregulate biosynthesis of antimicrobial phenolics in food and medicinal plants providing multilayered protection against several foodborne pathogens. Such blue or red light-mediated strategy is specifically effective for indoor vertical farming, especially to grow microgreens, micro-herbs, and sprouts with antimicrobial benefits.

Like in the case of non-white light, short exposure to UV treatment can also stimulate biosynthesis of phenolics with antimicrobial functionalities in food and medicinal plants. Previously, stimulation of proline-associated PPP regulation and enhanced phenolic-linked antioxidant enzyme activity were observed in fava bean (*Vicia faba*) [104], snow algae (*Chlamydomonas nivalis*) [105], and cool-season turfgrasses [106] with UV-C and UV-B treatment. Therefore, UV stress exposure is an effective strategy to improve stress-inducible phenolics in food and medicinal plants through the up-regulation of redox-linked PPP, and such a strategy can be targeted to improve the phenolic antimicrobials for potential food safety applications. In a study with radish sprouts, an accumulation of anthocyanins in the hypocotyls was observed after exposure to UV-B radiation for up to 48 h followed by incubation in dark conditions, and a positive correlation coefficient of 0.80 was found between anthocyanin content and PAL activity [30]. In another study, mango cultivar Haden was exposed to UV-C radiation at energy levels of 2.46 and 4.93 kJ m^−2^ to improve the postharvest quality, and it was found that UV-C treatment resulted in higher levels of phenolics and flavonoid content, along with an increase in PAL activity [107]. 

Controlled wounding is also another physical stress, which can induce adaptive defense response in fresh foods including enhanced biosynthesis of protective phenolics. In one study, carrot cultivars Navajo, Legend, and Choctaw were subjected to different wounding intensities (slices, pies, and shreds), and it was found that shredded carrots had a ~2.5 fold increase in soluble phenolics, when compared to whole carrots, as well as a ~9.5-, 7.3-, and 11.3-fold increase in chlorogenic acid content and a ~19.2-, 27.9- and 266.2-fold increase in PAL activity for the shredded Navajo, Legend, and Choctaw cultivars, respectively, when compared to whole carrots (*p* < 0.05) [108]. In the same study, ferulic acid was detected only in the wounded carrot and not in the whole carrots [108]. Gamma radiation is another strategy that can be used to enhance the production of secondary metabolites such as phenolics and flavonoids, which can be attributed to the increase in activity of the enzymes PAL and CHS that are involved in the biosynthetic pathways for the production of phenolic acids and flavonoids, respectively [23,109,110,111]. Therefore, these physical elicitors can be rationally targeted to stimulate stress-adaptive responses such as anabolic PPP regulation for enhancing the biosynthesis and bioactivity of stress-inducible phenolics, specifically for antimicrobial benefits relevant for food safety applications.

### 6.2. Chemical Elicitation Strategies

Biotic and abiotic chemical elicitors such as lipopolysaccharides, plant herbal extracts, peptides, and organic acids can stimulate the biosynthesis of stress-inducible phenolics in treated seeds resulting in enhanced phenolic phytochemical content and bioactive properties such as antioxidant and antimicrobial activity [85,112]. Seeds of dark germinated mung bean treated with extracts of the oregano clonal line O-4: E enriched in rosmarinic acid showed higher total phenolic content and antioxidant activity, when compared to the control seeds that were treated with distilled water [112]. Dark germinated mung bean seeds treated with lactoferrin (peptide elicitor) or oregano extract (herbal elicitor) showed higher antimicrobial activity against *H. pylori* during the early stages of germination [85]. In the same study, stimulation of proline-associated PPP and subsequent increase in phenolic content was also observed with natural elicitor treatments [85]. Hairy root cultures of *Plantago lanceolata* transformed with *Agrobacterium tumefaciens* and treated with AgNO_3_ (20 mg·L^−1^) or chitosan (100 mg·L^−1^) showed a 7.63- and 4.76-fold increase in gallic acid content, respectively, after 24 h of incubation, when compared to the untreated hairy root cultures [86]. The same study showed the methanolic extracts of hairy root cultures treated with AgNO_3_ to have a MIC of 25 mg mL^−1^ against *K. pnuemoniae*, *P. vulgaris,* and *S. typhi* [86]. 

Pre-harvest treatment with ozone sprayed at the rate of 25 gallons per acre, once every ten days for a sixty-day period, resulted in higher total soluble phenolic content in the red and white grape cultivars, Frontenac and Vignoles respectively, when compared to the control (untreated) grapes [28]. In another study, ethanolic extracts of grape skins from 14 different varieties (white and red grape) showed antimicrobial activity against Gram-positive (*S. aureus* and *B. cereus*) and Gram-negative (*E. coli* and *S. infantis*) bacterial pathogens with a MIC ranging from 0.014 to 0.59 mg gallic acid equivalents (GAE) per mL [113]. Furthermore, the same study found that, on average, the white grape varieties gave a lower MIC than red grape varieties, and that *E. coli*, *S. infantis,* and *B. cereus* were the most susceptible due to their lower MICs [113]. Roots of hydroponically grown plants were treated with 0.1% acetate and 0.1% chitosan, and it was found that the number of plant species that displayed antimicrobial activity against *S. aureus* subsp. *aureus*, *E. coli* K12, and *P. aeruginosa* were doubled after treatment with these elicitors [87].

In another study, fresh cut pineapples and bananas exposed to ozone at a flow rate of 8 ± 0.2 mL/s for 0, 10, 20, and 30 min showed the highest polyphenol and flavonoid content at 20 min of ozone exposure, and this was attributed to the increase in PAL activity [114]. Shoot tip explants of red cabbage with zeatin (2 mg/L) showed higher phenolic and flavonoid content, higher antioxidant activity, as well as higher cytotoxic activity against NIH-3T3 cancer cells during the elongated shoot stage when compared to the untreated (control) shoots [100]. Similarly, foliar application of COS at pre-harvest stage of Greek oregano at a concentration of 50 and 200 ppm was found to upregulate the polyphenolic content and induce H_2_O_2_ generation in the leaves [115]. Strawberries treated with chitosan solutions at post-harvest stage were found to have higher levels of phenolics, flavonoids, anthocyanins, and antioxidant enzyme activity as well as an improvement in shelf life when compared to the control (untreated) strawberries [116]. The improved biosynthesis of stress-inducible phenolics and associated functionalities in elicitor-treated plant-based foods are potentially relevant in providing protection against bacterial pathogens including foodborne pathogens. Such chemical elicitor-based innovations, especially with biologically derived elicitors, are a safe and effective strategy to improve human health as well as food safety related antimicrobial phenolics in food and medicinal plants. 

### 6.3. Microbial Elicitation Strategies

Biotic elicitors of microbial origin include compounds that are directly released by the microorganism (e.g., xanthan gum), or that are produced by action of the microorganism on plant cell wall (e.g., pectin), or by action of plant enzymes on the microbial cell wall (e.g., chitosans), and they have potential to stimulate the production of plant secondary metabolites such as phenolic bioactives [96]. Seeds of dark germinated mung beans treated with food-grade microbial polysaccharides (xantham gum and gellan gum), fungal polysaccharides (glucan and yeast extract), and acids (acetic acid and salicylic acid) had a higher phenolic content in the leaves and cotyledons at day 5 of germination, when compared to the control or untreated seeds [117]. Hairy root cultures of sweet basil (*Ocimum basilicum* L) transformed with *A. rhizogenes* showed a 3-fold increase in growth and rosmarinic acid production, when compared to the untransformed cultures, and rosmarinic acid also displayed antimicrobial activity against the *P. aeruginosa* strains PAO1 and PA14, which are opportunistic pathogens that can infect plants, animals, and humans [88]. In the same study the root cultures were treated with fungal cell wall elicitors isolated from the plant fungal pathogen *Phytophthora cinnamon,* and the treated cultures showed a 2.67-fold increase in rosmarinic acid, when compared to the untreated roots [88]. Soybean seedlings pre-exposed to a combined treatment of H_2_O_2_ or ROS with AgNO_3_ were treated with live preparations of the microbial elicitors *B. subtilis* or *Rhizopus* to improve the content and profile of prenylated isoflavonoids, which are phenolic compounds that display potential antimicrobial activity [118]. The study showed that the H_2_O_2_ + AgNO_3_ treated seedlings exposed to *B. subtilis* gave 30% more content in prenylated isoflavonoids when compared to H_2_O_2_ + AgNO_3_ treated seedlings that were exposed to *Rhizopus* [118]. Cell cultures of *Coleus blumei* treated with either a fungal elicitor (culture medium containing the phytopathogenic oomycete *Pythium aphanidermatum*) or with methyl jasmonate, showed an approximate 3-fold increase in rosmarinic acid content, and the fungal elicitor enhanced the activity of PAL and rosmarinic acid synthase, while methyl jasmonate transiently increased the activity of PAL and hydroxyphenylpyruvate reductase and also slightly enhanced the activity of tyrosine aminotransferase, which are key enzymes involved in the biosynthesis of rosmarinic acid [119]. Venus fly trap (*Dionaea muscipula* J. Ellis), an important medicinal herb, was elicited using a combination of biotic elicitation (*Cronobacter sakazakii* bacteria lysate) and physical elicitation (hydromechanical stress via rotary shaking) in order to improve the quantity and quality of phenolic compounds [89]. This study showed that the elicitation strategy led to an increased synthesis of myricetin, caffeic acid, and ellagic acid in *D. muscipula* J. Ellis tissue, and the minimal bactericidal concentration (MBC) against *S. aureus* was the lowest at 334 µg DW × mL^−1^ at day 7 of elicitation with 5% concentration of C. *sakazakii* lysate [89].

### 6.4. Phytohormone Elicitation Strategies

Plant hormones or phytohormones play a key role in the signaling pathways that help the plants to counterattack bacterial, fungal, and viral pathogens, and most common plant defense hormones are salicylic acid and jasmonic acid. Similarly, other phytohormones such as ethylene, abscisic acid, cytokinins, auxins, gibberellins, and brassinosteroids also help to modulate the stress-adaptive response of the plants against biotic stresses [120]. Salicylic acid (SA), a 2-hydroxybenzoic acid, is an important phytohormone involved in flower induction, thermogenesis, and allelopathic function and is produced via the phenylpropanoid pathway and/or via the isochorismate pathway [121,122]. SA plays a vital role as an endogenous signal inducing local and systemic acquired resistance in plants in response to a bacterial, viral, or fungal attack. The SA content in the infected leaves of tobacco mosaic virus (TMV) resistant tobacco was 50-fold higher compared to TMV-susceptible tobacco, while the uninfected leaves of the same TMV-resistant tobacco plant had a 10-fold higher SA content than those of TMV-susceptible tobacco [122]. Furthermore, the high SA levels in the TMV-resistant tobacco plant correlated with the presence of PR protein transcripts in both the infected and uninfected parts of the plant, and SA reached detectable levels 4 h after inoculation with *Pseudomonas syringae* pv. Syringae [122,123]. The local acquired resistance usually involves a hypersensitive response in which the infected plant cells undergo necrosis to contain the pathogen, which leads to the formation of plant lesions. Salicylic acid induces the expression of several plant defense-related genes during pathogen attack, which can lead to the strengthening of plant cell wall, the production of pathogenesis-related (PR) proteins such as antimicrobial enzymes and plant antimicrobial peptides (AMPs), as well as other secondary metabolites such as phytoalexins, all of which display a broad spectrum of antimicrobial activity [123,124]. 

Similarly, the phytohormone jasmonic acid and its derivative methyl jasmonate act as signaling molecules that activate transcription factors belonging to different families including AP2/ERF, bHLH, MYB, and WRKY, and the activated transcription factors bind to promoters of genes that code for regulatory enzymes involved in the biosynthesis of secondary metabolites such as antimicrobial phenolics [125]. In another study, roots of hydroponically grown plants were treated with 0.1 mM methyl jasmonate and 0.8 mM methyl salicylate, and antimicrobial activity against *S. aureus* subsp. *aureus*, *E. coli* K12, and *P. aeruginosa* was observed after treatment with these elicitors [87]. Dark germinated pea sprouts were treated with acetyl salicylic acid and analyzed for antimicrobial activity against *H. pylori,* and the concentrated phenolic extracts of the treated and untreated pea sprouts showed a similar dose-dependent antimicrobial activity against *H. pylori* at days 5 and 8 of germination; this activity was attributed to the total soluble phenolic content and polymerized phenolic compounds [90]. Using carrot (*Daucus carota*) as a model system, a subtractive cDNA library was created to elucidate the roles of reactive oxygen species (ROS), ethylene (ET), and jasmonic acid (JA) in response to wounding stress in which carrot shreds were treated with inhibitors against ROS biosynthesis, ET action, and JA biosynthesis (diphenyleneiodonium chloride, 1- methylcyclopropene and phenidone, respectively), as separate or combined treatments, and were compared to untreated (control) carrot shreds [126]. Over 335 unique expressed sequence tags were obtained in this study, and based on relative gene expression data the authors concluded that ROS and ET were simultaneously produced during wounding stress and that ET induces the biosynthesis of JA [126]. This study also concluded that ROS played a major role as the key signaling molecule in the wound-induced activation of primary and secondary metabolism, while ET and JA were found to modulate ROS levels and possibly affect accumulation of polyphenols such as 3-O-caffeoylquinic acid, 3,5-dicaffeoylquinic acid, and 4,5-dicaffeoylquinic acid, among other compounds [126]. The activation of the plant defense related genes can result in a systemic acquired response that displays resistance to a broad spectrum of bacterial, viral, and fungal pathogens, and the expression of PR protein related genes can occur as a result of cross-talk between SA-dependent and SA-independent pathways that involve other phytohormones such as jasmonic acid and ethylene [97,126]. Therefore, plant hormones in an optimized dose can be targeted as an effective elicitor-based treatment strategy to stimulate endogenous defense responses such as protective PPP regulation, especially to enhance antimicrobial phenolics in fresh plant-based foods for protection against foodborne pathogens.

### 6.5. Other Strategies

Apart from elicitation-based strategies, there are other approaches that can be recruited to improve the protective phenolic content and the associated antimicrobial activity against foodborne bacterial pathogens. One of these approaches includes plant tissue culture and micro-propagation techniques that can be used to enhance the stress-inducible phenolic metabolite content of various medicinal and non-medicinal plants, thereby improving their biological activity in terms of their antioxidant, antimicrobial, and cytotoxic activity [22,127]. In vitro cultures of chickpea explants originating from cotyledon, leaf, node, shoot, and root apices were subjected to callogenesis and organogenesis, and the total phenolic content (mg/g callus) was found to significantly increase from the main callus culture to the 10th callus subculture along with an increase in browning due to accumulation of polyphenolic compounds [128]. The free and bound flavonoid extracts from seeds and callus tissues of three cotton (*Gossypium*) species were analyzed for their antimicrobial activity against *B. cereus*, *E. coli*, *Proteus vulgaris P. aeruginosa*, *S. typhimurium*, *S. aureus*, and *S. epidermis,* and it was observed that, in general, the callus tissues showed better antimicrobial activity when compared to the seed extracts, and the antimicrobial activity against these pathogens was selective depending upon the cotton species [91]. In another study, ethanol extracts of *Cichorium pumilum* Jacq. callus cultures displayed antimicrobial activity against *S. aureus*, *K. pneumoniae*, *B. cereus*, *B. subtilis,* and *P. aeruginosa,* while extracts of the ex vitro plantlets also displayed antimicrobial activity against these pathogens, including *E. coli*, *Enterobacter aerogenes,* and *S. epidermis* [92]. Chloroform and methanol leaf extracts of elite lines of *Stevia rebaudiana* Bertoni developed via micro-propagation displayed antimicrobial activity against *E. coli*, *B. subtilis*, *S. mutans,* and *S. aureus* in a dose-dependent manner, and the antimicrobial activity was similar to that of the leaf extracts of *Stevia rebaudiana* Bertoni grown in vivo [93]. Similarly, petroleum ether extracts of micro-propagated *Tulbaghia violacea* had significantly higher phenolic, flavonoid, and saponin content when compared to the plants grown outdoors (*p* < 0.05), and the micro-propagated plants showed low MICs of 0.39, 1.56, 0.78, and 1.56 mg/mL against *B. subtilis*, *E. coli*, *K. pneumoniae,* and *S. aureus,* respectively [94]. Previously, significant antibacterial activity against *H. pylori* and *L. monocytogenes* was observed in extracts of high phenolic, thymol, and carvacol containing elite clonal lines of oregano (*Origanum vulgare* L.), which were developed using novel micro-propagation strategies [47,95]. Micro-propagation strategies have the potential to develop and select elite clonal lines of food and medicinal plants with higher content of stress-inducible antimicrobial phenolics. Genetic engineering can be another approach to improve the phenolic content and food safety of plant-based foods [129,130]. The increase in plant secondary metabolite production and phenolic content can be achieved through genetic engineering strategies that target important factors in the secondary metabolite biosynthetic pathway such as overcoming rate-limiting steps, reducing flux through competitive pathways, reducing catabolism of products, and overexpression of regulatory genes [129]. Plants can be genetically engineered to express bacterial genes that code for enzymes involved in the production of nutritionally relevant compounds [130]. Through *Agrobacterium*-mediated transformation, researchers were able to express plant and bacterial genes that code for enzymes involved in the β carotene biosynthetic pathway, to improve the carotenoid content in rice and flax [131,132]. The *ubi*C gene from *E. coli* that codes for chorismate pyruvate lyase, which converts chorismate to 4-hydroxybenzoate, was expressed in *Nicotiana tabacum* via *Agrobacterium*-mediated transformation [133]. The same study showed that the transgenic tobacco plants expressing the *ubi*C gene had a 4-hydroxybenzoate content that was greater than the content found in the untransformed tobacco by at least a factor of 1000 [133]. Transcription of genes involved in secondary metabolite biosynthetic pathways are controlled by a group of proteins called transcription factors that bind to *cis*-acting elements in the promoter and enhancer regions of the target gene and initiate the binding of RNA polymerase II and other proteins to form the preinitiation complex [134]. The transcription factors MYB and bHLH have been characterized in *Arabidopsis thaliana* and are responsible for the expression of genes involved in the plant secondary metabolism such as the phenylpropanoid and flavonoid biosynthetic pathways [134,135,136]. Genes that code for the enzymes PAL and CHS contain elements in their promoter regions that are activated by external stress stimuli or elicitation such as exposure to UV radiation, fungal elicitors, or wounding [137,138,139,140]. Genetic engineering of plants to improve their secondary metabolism offers exciting prospects to enhance the phenolic content and associated antimicrobial activity. In this regard, genetic engineering and micro-propagation strategies alone or in combination with physical, chemical, and microbial elicitation can be a novel approach to further enhance the antimicrobial phenolic content and subsequently improve the food safety benefits of plant-based fresh and processed foods. 

## 7. Conclusions and Future Directions

Due to the complexity of bacterial contamination of plant-based foods, especially nutritionally relevant fresh foods, it is important to develop safe and effective strategies to address these global food safety challenges. Additionally, with rapidly increasing resistance of foodborne bacterial pathogens against common antibiotics drugs, food safety solutions are becoming more and more challenging. Therefore, innovations in new antimicrobial strategies must be able to provide multilayered barriers and protections against complex biological contaminations, targeting solutions against common pathogenic bacterial contamination of foods. In this context, recruiting metabolically driven strategies to stimulate natural host defense responses, such as redox-linked pentose phosphate pathway (PPP) regulation, and concurrently enhancing inducible phenolic content of food and medicinal plants has diverse benefits such as (i) improving plants resilience against biotic and abiotic stresses; (ii) improving human health relevant nutritional qualities of plant-based foods; (iii) enhancing shelf-life and post-harvest preservation qualities; and (iv) improving food safety through protection against contamination of foods with bacterial pathogens. Specifically, such stimulation of stress-inducible and antimicrobial phenolics in food and medicinal plants using elicitation strategies offers an exciting approach to address the rising cases of foodborne-associated diseases and the increase in infections involving multidrug-resistant bacterial pathogens globally. 

There are many published studies on elicitation strategies that focus on improving the organoleptic properties and the phenolic content or profile of food plants and plant-based foods, with the goal of enhancing their shelf-life as well as other bioactive properties that have human health relevant benefits. However, there are only a limited number of studies that directly examine the effects of elicitation on enhancing stress-inducible phenolics and potential antimicrobial activity of plants and plant-based foods against human bacterial pathogens, with the goal of improving their food safety by reducing the risk of bacterial contamination. Furthermore, plant phenolic phytochemicals need a minimum inhibitory concentration (MIC) to display substantial antimicrobial activity, and the elicitation method commonly used may or may not be adequate to produce enough phenolic content to achieve these MICs. To address these concerns, future studies need to be advanced by using a combination of physical, chemical, microbial, tissue culture, and genetic engineering based tools and techniques to help achieve the desired level of phenolic phytochemical content needed to display these MICs. Additionally, it is important to address the common food safety issues of nutritionally relevant plants, especially plants that are relevant for both nutritional-linked food security and NCD-linked public health solutions.

In this context, the cultivation of microgreens and sprouts has gained popularity in recent times due to the relative ease of cultivation and the low capital input required to grow these nutritionally relevant fresh plant foods. However, there is significant concern about food safety of sprouts and microgreens, specifically due to their higher susceptibility to bacterial contamination. In this regard, advancing elicitation strategies on microgreens and sprouts as well as for field-grown vegetables and fruits is a safe approach to enhance their protective phenolic content and concurrently improve their antimicrobial activity. It is also important to understand the impact of the application of physical or chemical elicitors on the common and beneficial microflora that exist in the phyllosphere or rhizosphere of the plant in order to improve the efficacy of these elicitation strategies. Additionally, beneficial microorganisms that are widely distributed across the food chain can also be recruited as microbial elicitors to stimulate host plant defense responses for upregulating biosynthetic pathways of antimicrobial phenolics in food and medicinal plants. However, it is essential to optimize different elicitation strategies based on types of food and medicinal plants and how they respond under specific and controlled stress induction. 

Overall, bio-preservation of food, especially to improve safety and quality of food products using natural antimicrobial compounds, is gaining increasing attention from both the food industry and consumers. Therefore, targeting a metabolically driven and biologically based elicitation strategy to enhance stress-inducible phenolic metabolites with antimicrobial function is a safe and effective approach to address the growing demands of the food industry and consumers, especially for more holistic and integrated food safety solutions. Such elicitation strategies with potential multilayered protection even can be tailored or optimized for specific food plants or for targeted solutions against contamination of food with foodborne pathogens. The enhancement of stress-inducible and antimicrobial phenolic metabolites with a novel elicitation strategy is not only relevant for diverse food safety solutions, but also important for improving nutritional and post-harvest preservation qualities of plant-based foods. Such an improvement in nutritional qualities and antimicrobial properties of plant-based foods is important to address global nutritional insecurity, food safety, and NCD-linked public health challenges that coexist in communities across the globe. 

## Figures and Tables

**Table 1:** Plant extracts and their antimicrobial activity against human bacterial pathogens.

Plant Extracts	Antimicrobial Activity ^a,b^	Bacterial Pathogen	Reference
*Andromeda polifolia*	1–3 mm	*S. aureus* DSM 20231 ^c^	[42]
	1–3 mm	*E. coli* ATCC 8739 ^d^	
*Calluna vulgaris*	1–3 mm	*S. aureus* DSM 20231	
	1–3 mm	*E. coli* ATCC 8739	
*Epilobium angustifolium*	4–10 mm	*S. aureus* DSM 20231	
	4–10 mm	*E. coli* ATCC 8739	
*Filipendula ulmaria*	4–10 mm	*S. aureus* DSM 20231	
	4–10 mm	*E. coli* ATCC 8739	
*Lythrum salicaria*	1–3 mm	*S. aureus* DSM 20231	
	4–10 mm	*E. coli* ATCC 8739	
*Matricaria chamomilla*	1–3 mm	*S. aureus* DSM 20231	
	3–4 mm	*E. coli* ATCC 8739	
*Tanacetum vulgare*	n.a	*S. aureus* DSM 20231	
	3–4 mm	*E. coli* ATCC 8739	
*Thymus vulgaris*	1–3 mm	*S. aureus* DSM 20231	
	4–10 mm	*E. coli* ATCC 8739	
*Allium cepa*	1–3 mm	*S. aureus* DSM 20231	
	4–10 mm	*E. coli* ATCC 8739	
*Avena sativa*	1–3 mm	*S. aureus* DSM 20231	
	1–3 mm	*E. coli* ATCC 8739	
*Beta vulgaris var. rubra*	1–3 mm	*S. aureus* DSM 20231	
	1–3 mm	*E. coli* ATCC 8739	
*Betula pubescens*	4–10 mm	*S. aureus* DSM 20231	
	1–3 mm	*E. coli* ATCC 8739	
*Picea abies*	1–3 mm	*S. aureus* DSM 20231	
	n.a	*E. coli* ATCC 8739	
*Pinus sylvestris*	4–10 mm	*S. aureus* DSM 20231	
	1–3 mm	*E. coli* ATCC 8739	
*Salix caprea*	1–3 mm	*S. aureus* DSM 20231	
	1–3 mm	*E. coli* ATCC 8739	
*Secale cereale*	1–3 mm	*S. aureus* DSM 20231	
	1–3 mm	*E. coli* ATCC 8739	
*Solanum tuberosum*	3–4 mm	*S. aureus* DSM 20231	
	1–3 mm	*E. coli* ATCC 8739	
*Aronia melanocarpa*	1–3 mm	*S. aureus* DSM 20231	
	1–3 mm	*B. subtilis* ATCC 9372	
	4–10 mm	*M. luteus* YMBL ^e^	
	1–3 mm	*E. coli* ATCC 8739	
*Empetrum nigrum*	1–3 mm	*S. aureus* DSM 20231	
	1–3 mm	*S. epidermis* ATCC 12228	
	3–4 mm	*B. subtilis* ATCC 9372	
	4–10 mm	*M. luteus* YMBL	
	1–3 mm	*E. coli* ATCC 8739	
*Malus pumila*	1–3 mm	*S. aureus* DSM 20231	
	n.a	*S. epidermis* ATCC 12228	
	1–3 mm	*B. subtilis* ATCC 9372	
	n.a	*M. luteus* YMBL	
	n.a	*E. coli* ATCC 8739	
*Ribes nigrum*	1–3 mm	*S. aureus* DSM 20231	
	n.a	*S. epidermis* ATCC 12228	
	1–3 mm	*B. subtilis* ATCC 9372	
	3–4 mm	*M. luteus* YMBL	
	n.a	*E. coli* ATCC 8739	
*Rubus chamaemorus*	1–3 mm	*S. aureus* DSM 20231	
	3–4 mm	*S. epidermis* ATCC 12228	
	3–4 mm	*B. subtilis* ATCC 9372	
	1–3 mm	*M. luteus* YMBL	
	1–3 mm	*E. coli* ATCC 8739	
*Rubus idaeus*	1–3 mm	*S. aureus* DSM 20231	
	1–3 mm	*S. epidermis* ATCC 12228	
	3–4 mm	*B. subtilis* ATCC 9372	
	1–3 mm	*M. luteus* YMBL	
	1–3 mm	*E. coli* ATCC 8739	
*Sorbus aucuparia*	1–3 mm	*S. aureus* DSM 20231	
	n.a	*S. epidermis* ATCC 12228	
	1–3 mm	*B. subtilis* ATCC 9372	
	1–3 mm	*M. luteus* YMBL	
	n.a	*E. coli* ATCC 8739	
*Vaccinium myrtillus*	1–3 mm	*S. aureus* DSM 20231	
	1–3 mm	*S. epidermis* ATCC 12228	
	1–3 mm	*B. subtilis* ATCC 9372	
	4–10 mm	*M. luteus* YMBL	
	1–3 mm	*E. coli* ATCC 8739	
*Vaccinium oxycoccus*	3–4 mm	*S. aureus* DSM 20231	
	n.a	*S. epidermis* ATCC 12228	
	n.a	*B. subtilis* ATCC 9372	
	n.a	*M. luteus* YMBL	
	4–10 mm	*E. coli* ATCC 8739	
*Vaccinium uliginosum*	1–3 mm	*S. aureus* DSM 20231	
	1–3 mm	*S. epidermis* ATCC 12228	
	1–3 mm	*B. subtilis* ATCC 9372	
	1–3 mm	*M. luteus* YMBL	
	n.a	*E. coli* ATCC 8739	
*Vaccinium vitis-*idaea	n.a	*S. aureus* DSM 20231	
	n.a	*S. epidermis* ATCC 12228	
	1–3 mm	*B. subtilis* ATCC 9372	
	1–3 mm	*M. luteus* YMBL	
	n.a	*E. coli* ATCC 8739	
Tomato cv. Pitenza			[43]
Stem	10.0 mm	S. Typhimurium ATCC 14028	
	8.6 mm	*E. coli* O157:H7 ATCC 43890	
	10.3 mm	*S. aureus* ATCC 65384	
	11.1 mm	*L. ivanovii* ATCC 19119	
Leaf	10.8 mm	S. Typhimurium ATCC 14028	
	9.4 mm	*E. coli* O157:H7 ATCC 43890	
	11.3 mm	*S. aureus* ATCC 65384	
	12.9 mm	*L. ivanovii* ATCC 19119	
Root	8.7 mm	S. Typhimurium ATCC 14028	
	8.3 mm	*E. coli* O157:H7 ATCC 43890	
	9.0 mm	*S. aureus* ATCC 65384	
	8.0 mm	*L. ivanovii* ATCC 19119	
Whole plant	8.0 mm	S. Typhimurium ATCC 14028	
	8.2 mm	*E. coli* O157:H7 ATCC 43890	
	9.9 mm	*S. aureus* ATCC 65384	
	8.9 mm	*L. ivanovii* ATCC 19119	
Tomato cv. Floradade			
Stem	n.a	S. Typhimurium ATCC 14028	
	n.a	*E. coli* O157:H7 ATCC 43890	
	10.3 mm	*S. aureus* ATCC 65384	
	n.a	*L. ivanovii* ATCC 19119	
Leaf	10.0 mm	S. typhimurium ATCC 14028	
	9.4 mm	*E. coli* O157:H7 ATCC 43890	
	9.3 mm	*S. aureus* ATCC 65384	
	9.0 mm	*L. ivanovii* ATCC 19119	
Root	n.a	S. Typhimurium ATCC 14028	
	n.a	*E. coli* O157:H7 ATCC 43890	
	n.a	*S. aureus* ATCC 65384	
	n.a	*L. ivanovii* ATCC 19119	
Whole plant	n.a	S. Typhimurium ATCC 14028	
	7.7 mm	*E. coli* O157:H7 ATCC 43890	
	8.2 mm	*S. aureus* ATCC 65384	
	n.a	*L. ivanovii* ATCC 19119	
Cranberry	3.3 mg/mL	*H. pylori* NCTC 11637 ^f^	[44]
	3.3 mg/mL	*H. pylori* NCTC 11638	
Oregano-cranberry combination	1.4–2.5 mm	*L. monocytogenes* Scott A 4b ^g^	[45]
Oregano-cranberry combination	1.5–3.0 mm	*V. parahaemolyticus* ^g^	[46]
Oregano extracts	1.2 mg/mL	*L. monocytogenes* 4b ^g^	[47]
Sage	0.82 mg/mL	*C. coli* ATCC 33,559	[48]
	6.72 mg/mL	*E. coli* O157:H7 ZM370 ^h^	
	6.72 mg/mL	*Salmonella* Infantis ZM9	
	1.68 mg/mL	*B. cereus* WSBC 10530 ^i^	
	1.68 mg/mL	*L. monocytogenes* ZM58	
	0.34 mg/mL	*S. aureus* ATCC 25923	
Thyme	3.40 mg/mL	*C. coli* ATCC 33,559	
	6.73 mg/mL	*E. coli* O157:H7 ZM370	
	6.73 mg/mL	*Salmonella* Infantis ZM9	
	6.73 mg/mL	*B. cereus* WSBC 10530	
	6.73 mg/mL	*L. monocytogenes* ZM58	
	6.48 mg/mL	*S. aureus* ATCC 25923	
Lemon balm	3.40 mg/mL	*C. coli* ATCC 33,559	
	6.73 mg/mL	*E. coli* O157:H7 ZM370	
	6.73 mg/mL	*Salmonella* Infantis ZM9	
	6.73 mg/mL	*B. cereus* WSBC 10530	
	6.73 mg/mL	*L. monocytogenes* ZM58	
	2.43 mg/mL	*S. aureus* ATCC 25923	
Peppermint	1.71 mg/mL	*C. coli* ATCC 33,559	
	6.71 mg/mL	*E. coli* O157:H7 ZM370	
	6.71 mg/mL	*Salmonella* Infantis ZM9	
	6.71 mg/mL	*B. cereus* WSBC 10530	
	6.71 mg/mL	*L. monocytogenes* ZM58	
	6.85 mg/mL	*S. aureus* ATCC 25923	
Oregano	1.70 mg/mL	*C. coli* ATCC 33,559	
	6.72 mg/mL	*E. coli* O157:H7 ZM370	
	6.72 mg/mL	*Salmonella* Infantis ZM9	
	3.36 mg/mL	*B. cereus* WSBC 10530	
	6.72 mg/mL	*L. monocytogenes* ZM58	
	5.60 mg/mL	*S. aureus* ATCC 25923	
Sage	0.010 mg GAE/mL	*B. cereus* WSBC 10,530	[49]
	0.020 mg GAE/mL	*S. aureus* ATCC 25923	
	0.161 mg GAE/mL	*E. coli* O157:H7 ZM370	
	0.161 mg GAE/mL	*Salmonella* Infantis ZMJ9	
*Tamarindus indica*	0.6 mm	*E. coli* ^j^	[50]
	1.0 mm	*S. aureus* ^j^	
	0.8 mm	*P. aeruginosa* ^j^	
	n.a	*S. typhi* ^j^	
*Morinda citrifolia*			[51]
Leaf	10 mm	*S. aureus* ^k^	
	11 mm	*S. epidermis* ^k^	
	9 mm	*Streptococcus pyogenes* ^k^	
	9 mm	*E. coli* ^k^	
	10 mm	*Serratia marcescens* ^k^	
	11 mm	*P. aeruginosa* ^k^	
	8 mm	*K. pneumoniae* ^k^	
Stem	9 mm	*S. aureus*	
	9 mm	*S. epidermis*	
	8 mm	*Streptococcus pyogenes*	
	7 mm	*E. coli*	
	7 mm	*Serratia marcescens*	
	8 mm	*P. aeruginosa*	
	7 mm	*K. pneumoniae*	
Root	9 mm	*S. aureus*	
	12 mm	*S. epidermis*	
	11 mm	*Streptococcus pyogenes*	
	9 mm	*E. coli*	
	8 mm	*Serratia marcescens*	
	11 mm	*P. aeruginosa*	
	11 mm	*K. pneumoniae*	
*Aspilia mossambicensis* ^l^			[52]
Leaf	10–14 mm	*S. aureus* ^m^	
	10–14 mm	*P. aeruginosa* ATCC 27853	
Stem bark	10–14 mm	*S. aureus*	
	8–9 mm	*P. aeruginosa* ATCC 27853	
Root	8–9 mm	*S. aureus*	
	n.a	*P. aeruginosa* ATCC 27853	
*Ocimum gratissimum*			
Leaf	n.a	*S. aureus*	
	n.a	*P. aeruginosa* ATCC 27853	
Stem bark	10–14 mm	*S. aureus*	
	8–9 mm	*P. aeruginosa* ATCC 27853	
Root	10–14 mm	*S. aureus*	
	10–14 mm	*P. aeruginosa* ATCC 27853	
*Toddalia asiatica*			
Leaf	n.a	*S. aureus*	
	n.a	*P. aeruginosa* ATCC 27853	
Stem bark	15–19 mm	*S. aureus*	
	10–14 mm	*P. aeruginosa* ATCC 27853	
Root	10–14 mm	*S. aureus*	
	n.a	*P. aeruginosa* ATCC 27853	
*Ocimum gratissimum*	20 mm	*P. aeruginosa* ^n^	[53]
	29 mm	*S. dysenteriae*	
	8.5 mm	*Proteus* sp.	
	18 mm	*S. aureus*	
Black pearl purple corn	13.33 mm	*S. enteritidis* ATCC13076	[54]
	10.33 mm	*S. aureus* ATCC 6538	
	n.a	*E. coli* ATCC 11775	
Jinheiyu purple corn	11 mm	*S. enteritidis* ATCC13076	
	10.17 mm	*S. aureus* ATCC 6538	
	n.a	*E. coli* ATCC 11775	
Jingheinuo purple corn	11.5 mm	*S. enteritidis* ATCC13076	
	9.17 mm	*S. aureus* ATCC 6538	
	n.a	*E. coli* ATCC 11775	
Shijiazhuang purple corn	13.33 mm	*S. enteritidis* ATCC13076	
	12.17 mm	*S. aureus* ATCC 6538	
	n.a	*E. coli* ATCC 11775	
Zhuozhou purple corn	14.33 mm	*S. enteritidis* ATCC13076	
	12.33 mm	*S. aureus* ATCC 6538	
	n.a	*E. coli* ATCC 11775	

n.a, no antimicrobial activity detected. ^a^ Antimicrobial activity of the highest concentration of each plant extract is shown. ^b^ Antimicrobial activity expressed as zone of inhibition (mm) or as minimal inhibitory concentration (mg/mL). ^c^ Deutsche Sammlung von Micro-organismen, Germany. ^d^ American Type Culture Collection, USA. ^e^ Division of General Microbiology, University of Helsinki, Finland. ^f^ National Collection of Type Cultures, U.K. ^g^ Department of Food Science, University of Massachusetts, USA. ^h^ ZIM Collection of Industrial Microorganisms, Slovenia. ^i^ Weihenstephan Microbial Strain Collection, Germany. ^j^ Microbiology Department, Usmanu Danfodiyo University Teaching Hospital, Nigeria. ^k^ Source of culture unknown. ^l^ Antimicrobial activity of only methanolic extracts are shown. ^m^ Clinical isolate, methicillin resistant. ^n^ Ondo State Specialist Hospital, Nigeria.

**Table 2:** Plant phenolic compounds and their antimicrobial activity against human pathogens.

Phenolic Compound	Antimicrobial Activity ^a,b^	Bacterial Pathogen	Reference
Protocatechuic acid	2000 (µg/mL)	*P. aeruginosa* ATCC 15692 ^c^	[35]
	2000 (µg/mL)	*P. aeruginosa* PA01 ^d^	
	2000 (µg/mL)	*P. aeruginosa* PT121 ^d^	
	2000 (µg/mL)	*P. aeruginosa* DB5218 ^e^	
	2000 (µg/mL)	*P. aeruginosa* DR3062 ^e^	
	n.a	*S. aureus* DSM 2031 ^f^	[42]
	n.a	*S. epidermis* ATCC 12228	
	n.a	*S. epidermis* FOMK ^g^	
	n.a	*B. subtilis* ATCC 9372	
	n.a	*B. subtilis* ATCC 6633	
	3–4 mm	*M. luteus* YMBL ^h^	
	n.a	*E. coli* ATCC 8739	
	n.a	*E. coli* ATCC 11775	
	4–10 mm	*P. aeruginosa* ATCC 9027	
Gallic acid	2000 (µg/mL)	*P. aeruginosa* ATCC 15692 ^c^	[35]
	2000 (µg/mL)	*P. aeruginosa* PA01 ^d^	
	2000 (µg/mL)	*P. aeruginosa* PT121 ^d^	
	2000 (µg/mL)	*P. aeruginosa* DB5218 ^e^	
	2000 (µg/mL)	*P. aeruginosa* DR3062 ^e^	
	9.0 mm	*E. coli* CECT 434 ^i^	[55]
	10.0 mm	*P. aeruginosa* ATCC 10145	
	7.75 mm	*L. monocytogenes* ATCC 15313	
	8.0 mm	*S. aureus* CECT 976	
	14 mm	*H. pylori* ATCC 700392	[56]
	14 mm	*H. pylori* ATCC 43504	
	n.a	*S. aureus* DSM 2031 ^f^	[42]
	n.a	*S. epidermis* ATCC 12228	
	1–3 mm	*S. epidermis* FOMK ^g^	
	n.a	*B. subtilis* ATCC 9372	
	n.a	*B. subtilis* ATCC 6633	
	1–3 mm	*M. luteus* YMBL ^h^	
	n.a	*E. coli* ATCC 8739	
	n.a	*E. coli* ATCC 11775	
	4–10 mm	*P. aeruginosa* ATCC 9027	
Rutin	4000 (µg/mL)	*P. aeruginosa* ATCC 15692 ^c^	[35]
	4000 (µg/mL)	*P. aeruginosa* PA01 ^d^	
	4000 (µg/mL)	*P. aeruginosa* PT121 ^d^	
	4000 (µg/mL)	*P. aeruginosa* DB5218 ^e^	
	4000 (µg/mL)	*P. aeruginosa* DR3062 ^e^	
	0.01 mg/mL	*E. coli* O157:H7 ATCC 43895	[57]
	0.015 mg/mL	*E. coli* O157:H7 ATCC 35150	
	0.02 mg/mL	*S. paratyphi* UK Micro29 A ^j^	
	0.02 mg/mL	*S. cholerasuis* subsp*. cholerasuis* ATCC 10708	
	0.02 mg/mL	*S. Enteritidis,* UK(-) H^2^S ^j^	
Caffeic acid	9.50 mm	*E. coli* CECT 434 ^i^	[55]
	9.0 mm	*P. aeruginosa* ATCC 10145	
	10.3 mm	*L. monocytogenes* ATCC 15313	
	9.75 mm	*S. aureus* CECT 976	
	n.a	*S. aureus* DSM 2031 ^f^	[42]
	n.a	*S. epidermis* ATCC 12228	
	1–3 mm	*S. epidermis* FOMK ^g^	
	n.a	*B. subtilis* ATCC 9372	
	n.a	*B. subtilis* ATCC 6633	
	n.a	*M. luteus* YMBL ^h^	
	n.a	*E. coli* ATCC 8739	
	n.a	*E. coli* ATCC 11775	
	1–3 mm	*P. aeruginosa* ATCC 9027	
Chlorogenic acid	7.25 mm	*E. coli* CECT 434 ^i^	[55]
	8.25 mm	*P. aeruginosa* ATCC 10145	
	n.a	*L. monocytogenes* ATCC 15313	
	n.a	*S. aureus* CECT 976	
	0.02 mg/mL	*E. coli* O157:H7 ATCC 43895	[57]
	0.015 mg/mL	*E. coli* O157:H7 ATCC 35150	
	0.005 mg/mL	*S. paratyphi* UK Micro29A ^j^	
	0.015 mg/mL	*S. cholerasuis* subsp*. cholerasuis* ATCC 10708	
	0.02 mg/mL	*S. Enteritidis,* UK(-) H_2_S ^j^	
(-) Epicatechin	7.25 mm	*E. coli* CECT 434 ^i^	[55]
	6.50 mm	*P. aeruginosa* ATCC 10145	
	n.a	*L. monocytogenes* ATCC 15313	
	n.a	*S. aureus* CECT 976	
	0.02 mg/mL	*E. coli* O157:H7 ATCC 43895	[57]
	0.015 mg/mL	*E. coli* O157:H7 ATCC 35150	
	0.01 mg/mL	*S. paratyphi* UK Micro29A	
	0.02 mg/mL	*S. cholerasuis* subsp*. cholerasuis* ATCC 10708	
	0.02 mg/mL	*S. Enteritidis,* UK(-) H2S	
Catechin	11 mm	*H. pylori* ATCC 700392	[56]
	16 mm	*H. pylori* ATCC 43504	
	1–3 mm	*S. aureus* DSM 2031	[42]
	n.a	*S. epidermis* ATCC 12228	
	1–3 mm	*S. epidermis* FOMK	
	n.a	*B. subtilis* ATCC 9372	
	n.a	*B. subtilis* ATCC 6633	
	n.a	*M. luteus* YMBL	
	n.a	*E. coli* ATCC 8739	
	n.a	*E. coli* ATCC 11775	
	1–3 mm	*P. aeruginosa* ATCC 9027	
Quercetin	0.02 mg/mL	*E. coli* O157:H7 ATCC 43895	[57]
	0.005 mg/mL	*E. coli* O157:H7 ATCC 35150	
	0.02 mg/mL	*S. paratyphi* UK Micro29A	
	0.015 mg/mL	*S. cholerasuis* subsp*. cholerasuis* ATCC 10708	
	0.02 mg/mL	*S. Enteritidis,* UK(-) H_2_S	
	4–10 mm	*S. aureus* DSM 2031 ^f^	[42]
	4–10 mm	*S. epidermis* ATCC 12228	
	4–10 mm	*S. epidermis* FOMK ^g^	
	1–3 mm	*B. subtilis* ATCC 9372	
	1–3 mm	*B. subtilis* ATCC 6633	
	4–10 mm	*M. luteus* YMBL ^h^	
	1–3 mm	*E. coli* ATCC 8739	
	3–4 mm	*E. coli* ATCC 11775	
	1–3 mm	*P. aeruginosa* ATCC 9027	
Ellagic acid	4000 (µg/mL)	*P. aeruginosa* ATCC 15692 ^c^	[35]
	4000 (µg/mL)	*P. aeruginosa* PA01 ^d^	
	4000 (µg/mL)	*P. aeruginosa* PT121 ^d^	
	4000 (µg/mL)	*P. aeruginosa* DB5218 ^e^	
	4000 (µg/mL)	*P. aeruginosa* DR3062 ^e^	
Tyrosol	n.a	*E. coli* CECT 434 ^i^	[55]
	8.0 mm	*P. aeruginosa* ATCC 10145	
	n.a	*L. monocytogenes* ATCC 15313	
	n.a	*S. aureus* CECT 976	
Ferulic acid	9.25 mm	*E. coli* CECT 434	
	9.0 mm	*P. aeruginosa* ATCC 10145	
	11.5 mm	*L. monocytogenes* ATCC 15313	
	10.5 mm	*S. aureus* CECT 976	
Phloridzin	n.a	*E. coli* CECT 434	
	n.a	*P. aeruginosa* ATCC 10145	
	n.a	*L. monocytogenes* ATCC 15313	
	n.a	*S. aureus* CECT 976	
Epigallocatechin gallate/tetracycline combination	0.5 (µg/mL)	*S. epidermis* (resistant) ^k^	[58]
	0.5 (µg/mL)	*S. aureus* (resistant) ^l^	
	0.0625 (µg/mL)	*S. epidermis* (susceptible) ^k^	
	0.125 (µg/mL)	*S. aureus* (susceptible) ^l^	
Catechin- gallic acid combination	13 mm	*H. pylori* ATCC 700392	[56]
	11 mm	*H. pylori* ATCC 43504	
Curcumin	0.02 mg/mL	*E. coli* O157:H7 ATCC 43895	[57]
	0.005 mg/mL	*E. coli* O157:H7 ATCC 35150	
	0.015 mg/mL	*S. paratyphi* UK Micro29A ^j^	
	0.015 mg/mL	*S. cholerasuis* subsp*. cholerasuis* ATCC 10708	
	0.005 mg/mL	*S. Enteritidis,* UK(-) H_2_S ^j^	
Eugenol	0.02 mg/mL	*E. coli* O157:H7 ATCC 43895	
	0.02 mg/mL	*E. coli* O157:H7 ATCC 35150	
	0.02 mg/mL	*S. paratyphi* UK Micro29A	
	0.02 mg/mL	*S. cholerasuis* subsp*. cholerasuis* ATCC 10708	
	0.015 mg/mL	*S. Enteritidis*, UK(-) H_2_S	
Myricetin	0.01 mg/mL	*E. coli* O157:H7 ATCC 43895	
	0.01 mg/mL	*E. coli* O157:H7 ATCC 35150	
	0.015 mg/mL	*S. paratyphi* UK Micro29A	
	0.02 mg/mL	*S. cholerasuis* subsp*. cholerasuis* ATCC 10708	
	0.01 mg/mL	*S. Enteritidis,* UK(-) H_2_S	
Flavone	4–10 mm	*S. aureus* DSM 2031 ^f^	[42]
	1–3 mm	*S. epidermis* ATCC 12228	
	4–10 mm	*S. epidermis* FOMK ^g^	
	4–10 mm	*B. subtilis* ATCC 9372	
	3–4 mm	*B. subtilis* ATCC 6633	
	>10 mm	*M. luteus* YMBL ^h^	
	3–4 mm	*E. coli* ATCC 8739	
	4–10 mm	*E. coli* ATCC 11775	
	1–3 mm	*P. aeruginosa* ATCC 9027	
Naringenin	>10 mm	*S. aureus* DSM 2031	
	>10 mm	*S. epidermis* ATCC 12228	
	>10 mm	*S. epidermis* FOMK	
	>10 mm	*B. subtilis* ATCC 9372	
	4–10 mm	*B. subtilis* ATCC 6633	
	>10 mm	*M. luteus* YMBL	
	3–4 mm	*E. coli* ATCC 8739	
	4–10 mm	*E. coli* ATCC 11775	
	1–3 mm	*P. aeruginosa* ATCC 9027	
Naringin	n.a	*S. aureus* DSM 2031	
	n.a	*S. epidermis* ATCC 12228	
	n.a	*S. epidermis* FOMK	
	n.a	*B. subtilis* ATCC 9372	
	n.a	*B. subtilis* ATCC 6633	
	4–10 mm	*M. luteus* YMBL	
	n.a	*E. coli* ATCC 8739	
	n.a	*E. coli* ATCC 11775	
	4–10 mm	*P. aeruginosa* ATCC 9027	
Methyl gallate	n.a	*S. aureus* DSM 2031	
	n.a	*S. epidermis* ATCC 12228	
	n.t	*S. epidermis* FOMK	
	n.a	*B. subtilis* ATCC 9372	
	n.t	*B. subtilis* ATCC 6633	
	>10 mm	*M. luteus* YMBL	
	4–10 mm	*E. coli* ATCC 8739	
	n.t	*E. coli* ATCC 11775	
	n.t	*P. aeruginosa* ATCC 9027	

n.a, no antimicrobial activity detected. n.t, not tested. ^a^ Antimicrobial activity of the highest concentration of each phenolic compound is shown. ^b^ Antimicrobial activity expressed as zone of inhibition (mm) or as minimal inhibitory concentration (mg/mL). ^c^ American Type Culture Collection. ^d^ University of Geneva, Switzerland. ^e^ Singapore General Hospital, Singapore. ^f^ Deutsche Sammlung von Micro-organismen, Germany. ^g^ Division of Pharmacognosy, University of Helsinki, Finland. ^h^ Division of General Microbiology, University of Helsinki, Finland. ^i^ Spanish Type Culture Collection, Spain. ^j^ University of Kentucky, USA. ^k^ Tetracycline resistant, private collection. ^l^ Tetracycline susceptible, private collection.

**Table 3:** Elicitation strategies to enhance phytochemical content and associated antimicrobial activity.

Elicitor/Other Strategy Used	Target Plant/Plant Family	Phytochemicals Analyzed ^a^	Target Bacterial Pathogen	References
Blue light	Spinach	Caffeic acid	*E. coli* O157:H7 ATCC ^b^ 700,728 *L. innocua* ^c^	[83]
Light or dark incubated callus culture.	Black mustard	Flavonoids, tannins & volatile oils.	*E. coli* ATCC 11229 *P. aeruginosa* ^d^ *K. pneumoniae* ATCC 13,883 *S. aureus* NCTC 7447 ^e^	[84]
Fish protein hydrolysate, oregano extract & lactoferrin.	Mung bean	Total phenolics	*H. pylori* ATCC 43579	[85]
AgNO_3_ & chitosan	*Plantago lanceolata*	Apigenin & gallic acid	*B. cereus* ^f^ *K. pneumoniae* ^f^ *P. vulgaris* ^f^ *S. typhi* ^f^ *E. coli* ^f^	[86]
Acetate, chitosan, methyl salicylate & methyl jasmonate	Anacardiaceae, Apiaceae, Asteraceae, Brassicaceae, Caryophyllaceae, Cucurbitaceae, Fabaceae, Lamiaceae, Polemoniaceae	n.t	*S. aureus* subsp. *aureus* ATCC 6538 *E. coli K12* ^d^ *P. aeruginosa* ^d^	[87]
Transformation with *A. rhizogenes* ATCC 15,834 *&* fungal elicitors.	Sweet basil	Rosmarinic acid	*P. aeruginosa* PAO1 ^g^ *P. aeruginosa* PA14 ^g^	[88]
*C. sakazakii* bacteria lysate & hydromechanical stress.	*D. muscipula* J. Ellis	Myricetin, caffeic acid, & ellagic acid	*S. aureus* ATCC 25923 *E. coli* ATCC 25922	[89]
Acetyl salicylic acid	Peas	Total phenolics	*H. pylori* ATCC 43579	[90]
Callus culture	Cotton	Free & bound flavonoids	*B. cereus* NCIM 2156 ^h^ *S. aureus* NCIM 2654 *S. epidermidis* NCIM 249 *M. smegmatis* NCIM 5138 *P. aeruginosa* NCIM 5032 *Proteus vulgaris* NCIM 2027 *S. typhimurium* NCIM 2501 *E. coli* NCIM 2027	[91]
Callus culture & ex vitro plantlets	Chicory	Total phenolics	*S. aureus* ATCC 29213 *K. pneumoniae* ATCC 10,031 *B. cereus* ATCC 11778 *B. subtilis* ATCC 6633 *P. aeruginosa* ATCC 27853 *E. coli* ATCC 25922 *E. aerogenes* ATCC 13048 *S. epidermis* ATCC 12,228 *Serratia marcescens* ATCC 27117	[92]
Micropropagation	*Stevia rebaudiana* Bertoni	n.t	*E. coli* MTCC 41 ^i^ *B. subtilis* MTCC 441 *S. mutans* MTCC 497 *S. aureus* MTCC 737	[93]
Micropropagation	*Tulbaghia violacea* Harv.	Total phenolics, flavonoids & saponins	*B. subtilis* ATCC 6051 *E. coli* ATCC 11775 *K. pneumoniae* ATCC 13,883 *S. aureus* ATCC 12600.	[94]
Micropropagation	Oregano	Carvacrol & thymol	*L. monocytogenes* ^j^	[47]
Micropropagation	Oregano	Total phenolics	*H. pylori* ATCC 43504	[95]

n.t, not tested. ^a^ Phytochemicals tested or detected in plant extracts. ^b^ American Type Culture Collection. ^c^ Private collection ^d^ Source of culture unknown. ^e^ National Collection of Type Cultures, U.K. ^f^ Iranian Biological Resource Center. ^g^ Dr. Herbert P. Schweizer’s laboratory, Colorado State University, USA. ^h^ National collection of industrial microorganisms, India. ^i^ Microbial Type Culture Collection, India. ^j^ Department of Food Science, University of Massachusetts, USA.

## Data Availability

No new data were created or analyzed in this study. Data sharing is not applicable to this article.
